# T82. THE RELATIONSHIP BETWEEN SOURCE MONITORING DEFICITS AND NEUROPSYCHOLOGICAL FUNCTIONING IN SCHIZOPHRENIA

**DOI:** 10.1093/schbul/sby016.358

**Published:** 2018-04-01

**Authors:** Martyna Krężołek, Łukasz Gawęda

**Affiliations:** 1 Medical University of Warsaw; 2 Medical University of Warsaw, University Medical Center Hamburg Eppendorf

## Abstract

**Background:**

Source monitoring (SM) is a metacognitive process involved in making judgments about the origin of memories, knowledge and beliefs. Many studies have demonstrated that people with schizophrenia perform more poorly on tasks of source monitoring when compared to non-schizophrenic. Although source of monitoring is considered as an important cognitive biases implicated in reality distortions/psychotic symptoms, the knowledge on its neurocognitive mechanisms is far from being conclusive. The main aim of our study was to investigate the relationship between SM and neuropsychological functioning in schizophrenia.

**Methods:**

A total of 84 (43 females; mean age 42.01, SD=11.55) patients diagnosed with schizophrenia were assessed with neuropsychological tests, including executive functions, verbal memory, working memory, processing speed and attention. SM was assessed with an action memory task. Simple actions were presented to the participant verbally (text) or non-verbally (icons). Some actions were physically performed and others were imagined. Following the learning phase, participants were presented with each action as well as new ones, were asked whether the action was presented verbally or non-verbally (action’s presentation type discrimination), and whether the action was performed or imagined (self-monitoring). A knowledge corruption for self-monitoring (proportion of high confident errors on all high confident responses) was also obtained. The symptoms severity was assessed with the PANSS. The relationship between SM biases and neuropsychological functioning was investigated with correlation analyses.

**Results:**

The correlations were found between incorrect action’s presentation type discrimination and the results of test such as CTT 1 (r=-0.22, p<0.05), D2 (r=-0.25, p<0.05) and Block Design (r= -0.40, p<0.01). Correlational analyses showed no relations between incorrect self-monitoring and neuropsychological functioning. Knowledge corruption for self-monitoring turned out to be correlated with WCST (r=0,22, p<0.05), CVLT (r= -0.26, p<0.05) and Backward Digit Span (r= -0.27, p<0.05). These correlations remain significant when controlled for positive symptoms severity. Incorrect self-monitoring showed a significant relation with the PANSS positive subscale (r=0.23, p<0.05). Knowledge corruption was related to PANSS disorganization subscale (r=0.25, p<0.05).

**Discussion:**

In line with previous studies we found that deficits in self-monitoring are related to symptoms severity and not to neuropsychological functioning. On the other hand, deficits in action’s presentation discrimination are related exclusively to neuropsychological functioning. These results suggest that the relationship between SM and neuropsychological functioning depends on the type of SM deficits. The conclusions of the study may be of clinical importance - in light of our results it might be advisable to combine cognitive remediation techniques with those interventions that focus on cognitive biases like source monitoring deficits

